# Psychiatric Diagnoses, Medication and Risk for Disability Pension in Multiple Sclerosis Patients; a Population-Based Register Study

**DOI:** 10.1371/journal.pone.0104165

**Published:** 2014-08-05

**Authors:** Philip Brenner, Kristina Alexanderson, Charlotte Björkenstam, Jan Hillert, Jussi Jokinen, Ellenor Mittendorfer-Rutz, Petter Tinghög

**Affiliations:** 1 Division of Psychiatry, Department of Clinical Neuroscience, Karolinska Institutet, Karolinska University Hospital, Stockholm, Sweden; 2 Division of Insurance Medicine, Department of Clinical Neuroscience, Karolinska Institutet, Stockholm, Sweden; 3 Division of Neuro, Department of Clinical Neuroscience, Karolinska Institutet, Karolinska University Hospital, Stockholm, Sweden; University of Oxford, United Kingdom

## Abstract

**Background:**

Psychiatric comorbidity is common among multiple sclerosis (MS) patients. The majority of MS patients of working ages are on disability pension. The aims of this study were to chart the prevalences of psychiatric diagnoses and medications among MS patients of working ages, and to investigate their association with the risk for future disability pension.

**Methods:**

This nationwide, population-based prospective cohort study includes 10,750 MS patients and 5,553,141 non-MS individuals who in 2005 were aged 17–64 years. Psychiatric diagnoses and medications were identified using nationwide registers. Odds ratios (ORs) with 95% confidence intervals (CIs) were calculated adjusting for socio-demographics. Furthermore, a survival analysis with five-year follow-up was performed among the 4,571 MS patients not on disability pension in 2005, with psychiatric diagnoses and medication as risk factors, and disability pension as the outcome.

**Results:**

Among MS patients, 35% had been prescribed psychiatric medication compared to 10% of non-MS individuals, adjusted OR 3.72 (95% CI 3.57 to 3.88). Ten percent of MS patients had received a psychiatric diagnosis, compared to 5.7% of non-MS individuals, OR 1.82 (95% CI 1.71 to 1.94). Serotonin reuptake inhibitors (SSRIs), were the most commonly prescribed drugs (17%) among MS patients, while depression (4.8%) was the most common psychiatric diagnosis. In the survival analysis, MS patients with any psychiatric diagnosis had a hazard ratio (HR) of 1.83 (95% CI 1.53 to 2.18) for disability pension compared to other MS patients. MS patients with any psychiatric drug prescription had a HR for disability pension of 2.09 (95% CI 1.84 to 2.33).

**Conclusion:**

Psychiatric diagnoses and medications are common among MS patients and adversely affect risk for disability pension. This highlights the importance of correct diagnosis and management of psychiatric comorbidity, in a clinical as well as in a societal perspective.

## Introduction

Multiple sclerosis (MS) is a neuroinflammatory disease which can affect all functions of the central nervous system. Various socio-demographic factors are associated with a higher risk for MS, such as female sex [1], place of residence during childhood [2], and low educational level [3]. MS is the most common cause of non-traumatic disability among young and middle-aged adults in the western hemisphere, and as such, leads to high rates of disability and societal costs [4]–[6]. In a recent study, the rate of MS patients of working ages who were disability pensioned was more than 60% [7], highly elevated compared to in the equivalent general population.

Psychiatric comorbidity is highly prevalent in MS patients, and is among the most important factors affecting quality of life, overall health, and disability [8]–[10]. Disability pension has been suggested as a reliable measure of social consequences of MS [11], and among MS patients of working ages, psychiatric comorbidity has been reported to increase the risk for disability pension [12]. However, the association of the risk for disability pension with having a specific psychiatric disorder or with being prescribed psychiatric drugs has, to the best of our knowledge, never been investigated.

So far, major depressive disorder (MDD) is the most investigated psychiatric disorder in MS patients. In a large Canadian survey, the rate of depression among MS patients was found to be around 14%, double that of the general population [13]. In MS patients attending hospital-based clinics, the life time risk of MDD has been reported to be as high as 50% [14], [15], with a point prevalence of 14%. Also, MDD is associated with higher levels of self-reported disability [9]. The risk for suicide, the most severe outcome of MDD, is more than doubled among MS patients compared to the general population [16].

It has been suggested that depressed MS patients do not receive adequate diagnosis and treatment. In a recent survey study, 48% of MS patients reported psychiatric comorbidity and an additional 16% fulfilled criteria for an undiagnosed depression [17]. In a sample of MS patients who were treated by neurologists, only 27% of the patients who met the criteria for MDD received pharmacological treatment at threshold levels, while 66% received no treatment at all [18].

The lifetime prevalence of anxiety disorders in MS patients is reported to be as high as 35%, with a point prevalence of 14% [19]. The risk for bipolar disorder seems to be about doubled in MS patients [20], with reported prevalence numbers ranging between 0.3% [21] and 13% [15] depending on the sample studied. The prevalence of psychotic disorders in MS patients was 0.8% in a population-based study, somewhat elevated compared to the general population [22]. Changes in personality traits are common in MS patients and have been shown to negatively affect quality of life and employment [23], but prevalence of formal personality disorder diagnoses has never been investigated.

Although some treatment guidelines do exist for these conditions, it has been largely uninvestigated what treatment MS patients actually do receive with regard to psychiatric disorders, as well as how different kinds of psychiatric comorbidity impact future work disability.

The aims of this study were to examine: a) the adjusted risk for different psychiatric diagnoses and medications among MS patients of working age compared to non-MS individuals, and b) whether presence of these diagnoses and medications are associated with future disability pension in MS patients.

## Materials and Methods

### Ethics

This study was approved by the Regional Ethics Committee, Stockholm Sweden (approval no. 2007/1762-31).

### Study population

In this population-based prospective cohort study, all individuals who were 17–64 years old and lived in Sweden in 2005 were selected from the Longitudinal Integration Database for Health Insurance and Labor Market Studies (LISA). This population-based register is held by Statistics Sweden and contains several demographic variables. After exclusion of 65,091 individuals (of which 41 were MS patients) with missing values on place of birth and/or educational level at baseline, the cohort consisted of 10,750 MS-patients, identified through nationwide registers, and 5,553,141 non-MS individuals. The subjects' unique personal identity numbers assigned to all Swedish residents were used to link data from different nationwide Swedish registers.

### Covariates

Information for 2005 regarding age-groups (17–34, 35–44, 45–54, 55–64), sex, educational level (compulsory school (≤9 years), high school (10–12 years), university (≥13 years), country of birth (Sweden, the other Nordic countries, other EU 25, all other countries), and type of living area (based on the H-region classification scheme [24] into the following three categories: larger cities (H1–H2), medium-sized (H3–H4) municipalities or smaller municipalities (H5–H6)) was obtained from LISA.

### Exposures and outcome variables

Diagnosis-specific data on MS, psychiatric hospitalization and specialized psychiatric out-patient care between 2000 and 2005 was obtained from the National Patient Register, held by the National Board of Health and Welfare. This register contains information on in-patient care since 1964 and specialized out-patient care since 2001. Presence of MS was defined as being hospitalized or receiving specialized care at least once between 2000 and 2005 with a primary or secondary diagnosis of the International Statistical Classification of Diseases and Related Health Problems, 10^th^ revision (ICD-10) [25] code G35. Psychiatric comorbidity was identified using ICD codes F00–F99, here organized into nine diagnostic categories; Developmental and organic disorders (F00–09, F70–F89), Disorders related to substance abuse (F10–F19), Schizophrenia and non-affective psychoses (F20–29), Bipolar disorder (F31), Depressive disorder (F32–F33), Affective disorders (F30–39, including bipolar disorder and depressive disorder), Neurotic and somatoform disorders (F40–F42, F44–F49), Stress-related mental disorders (F43), Behavioral disorders (F50–F59, F90–99), and Personality disorders (F60–F69).

The individuals were linked to data from the Swedish Prescribed Drug Register, which contains information on all prescribed drugs dispensed at a pharmacy in Sweden, to obtain information on all prescribed and purchased psychiatric medication from July to December 2005 (the register started July 1st 2005). Psychiatric medication was defined as having purchased drugs with the Anatomical Therapeutic Chemical (ATC) [26] codes N03–N07 at least once between July and December 2005. The codes were organized into nine different groups according to pharmacological class and/or clinical use: Selective Serotonin Reuptake Inhibitors (SSRIs; N06AB), Tricyclic antidepressants (TCAs; N06AA), other antidepressants (AD; N06AG02, N06AX), First generation antipsychotics (FGA; N05AA–N05AD, N05AF), Second and third generation antipsychotics (SGA); N05AE, N05AH0, N05AX), Benzodiazepines (N03AE01, N05BA, N05CD), Short-acting sleeping agents (N05CF), Lithium (N05AN01), and Alcohol dependence drugs (N07BB). Anti-convulsants and central stimulants were not included as their indications in MS patients were deemed dominantly somatic.

Information on being granted disability pension, the outcome measure, or old age pension in 2006–2010 was obtained from the national Social Insurance Agency's MiDAS database. In Sweden, all adult residents with a disease or injury that has led to permanent work incapacity can be granted disability pension. Disability pension, which may be granted for part- and full-time, covers up to 64% of the income lost. The customary age for old age pension is 65 years of, but may be granted earlier [27]. Date of death was obtained from the Cause of Death Register.

### Analysis

First, descriptive analyses were performed to explore the distribution of the baseline covariates among all MS patients and the non-MS individuals, respectively. Second, logistic regression was used to calculate odds ratios (ORs) with 95% confidence intervals (CI) for psychiatric in- and out-patient care (2001–2005) and for psychiatric drug prescriptions (July–December 2005), for MS-patients compared to non-MS individuals. The analyses were adjusted for age group, sex, educational level, country of birth, and type of living area.

Third, among the 4571 MS patients not on disability pension in 2005, survival analyses were performed with psychiatric diagnoses and medication as exposures, and disability pension as the outcome. The cohort members were followed from 2006 through 2010 or the year the individual turned 65, emigrated, died, or received old age or disability pension, whichever occured first. In each analysis, the reference group included all subjects without the exposure tested, i.e. included all other diagnosis groups and/or medications. Hazard ratios (HRs) with 95% confidence intervals (CI) were estimated by proportional hazards models. The analyses were adjusted for age group, sex, educational level, country of birth, and type of living area. The assumption of proportional hazards was tested and met using log/(−log) plots Each diagnosis group and prescribed drug class were analyzed separately throughout the study, not taking into account any co-occurrence of other diagnoses or drugs in the subjects.

Fourth, two-way interactions between age/sex and the exposure variable were introduced in all analyses to evaluate the fit of the models. These interactions were either deemed non-significant, or did only affect the results marginally, suggesting that all models fitted the data adequately. Furthermore, correction for multiple comparisons was performed using the Holm-Bonferroni method [28].

## Results

Table 1 shows base-line characteristic data for MS patients and non-MS individuals, respectively. MS patients were generally older, with a mean age of 47 vs 41 years, and more often female, 71% vs 49%. Disability pension was clearly more common among MS patients, 61% compared to 10% among non-MS individuals. A larger proportion of MS patients were born in Sweden, 91% compared to 86%. Other socio-demographic data did not differ substantially.

**Table 1:** Descriptive data of the studied cohort of multiple sclerosis (MS) patients and non-MS individuals aged 17–64 who lived in Sweden in 2005.

MS (n = 10,750)			Non-MS (n = 5,553,141)	
**Age (mean years, ±SD** 1 **)**	47.0±11.0		41.0±13.6	
**Age groups**	*n*	*(%)*	*n*	*%*
17–25	247	(2.3)	836,497	(15.1)
26–35	1,421	(13.2)	1,120,245	(20.2)
36–45	2,582	(24.0)	1,255,065	(22.6)
46–55	3,185	(29.6)	1,150,397	(20.7)
56–65	3,315	(30.8)	1,190,937	(21.4)
**Sex**				
Male	3,143	(29.2)	2,819,244	(50.7)
Female	7,607	(70.8)	2,733,897	(49.2)
**Disability pension**				
Yes	6,525	(60.7)	577,527	(10.4)
No	4,225	(39.3)	4,975,614	(89.6)
**Educational level**				
Compulsory School (≤9 years)	1,842	(17.1)	1,122,274	(20.2)
High school (10–12 years)	5,282	(49.1)	2,654,085	(47.8)
University (≥13 years)	3,626	(33.7)	1,776,782	(32)
**Country of birth**				
Sweden	9,772	(90.9)	4,767,541	(85.9)
Other Nordic countries	308	(2.9)	174,782	(3.1)
Other EU 25 countries	213	(2.0)	116,250	(2.1)
Other world countries	457	(4.3)	494,568	(8.9)
**Type of living area**				
Larger cities	3,960	(36.8)	2,058,354	(37.1)
Medium-sized municipalities	3,801	(35.4)	1,965,140	(35.4)
Smaller municipalities	2,989	(27.8)	1,529,647	(27.5)

1 SD = standard deviation.

In table 2, numbers and percentages of individuals with a psychiatric diagnosis or medication are shown for MS patients and non-MS individuals, respectively. It also shows the results of the logistic regression analysis presented as adjusted ORs. Ten percent of MS patients had received a psychiatric diagnosis, compared to 5.7% of non-MS individuals (OR 1.82 (95% CI 1.71–1.94)). Depressive disorder was the most common diagnosis in both groups; 4.4% of MS patients and 1.7% of the non-MS individuals, respectively (OR 2.41 (95% CI 2.22–2.64)). All psychiatric diagnoses were overrepresented in MS patients, except for personality disorders and substance abuse for which rates were approximately equal.

**Table 2:** Psychiatric diagnoses and medications in multiple sclerosis (MS) patients and non-MS individuals; absolute numbers, percentages and adjusted odds ratios (ORs) with 95% confidence intervals (CI).

	MS (n = 10,750)		Non-MS (n = 5,553,141)			
Diagnoses	n	(%)	n	(%)	OR^a^	(95%CI)^a^
Developmental, organic disorders	148	(1.4)	17,778	(0.3)	4.66	(3.96–5.49)*
Substance abuse	162	(1.5)	99,474	(1.8)	0.94	(0.81–1.10)
Psychotic disorders	82	(0.8)	31,728	(0.6)	1.25	(1.01–1.56)
Depressive disorders	471	(4.4)	95,275	(1.7)	2.41	(2.22–2.64)*
Bipolar disorder	51	(0.5)	12,072	(0.2)	1.73	(1.31–2.27)*
Affective disorders	542	(5.0)	109,969	(2.0)	2.37	(2.18–2.59)*
Neurotic, somatoform disorders	303	(2.8)	88,768	(1.6)	1.67	(1.49–1.88)*
Stress-related disorder	170	(1.6)	52,244	(0.9)	1.65	(1.42–1.92)*
Behavioral disorders	85	(0.8)	32,492	(0.6)	1.68	(1.36–2.08)*
Personality disorders	42	(0. 4)	22,088	(0.4)	0.98	(0.72–1.33)
Any psychiatric diagnosis	1074	(10.0)	317,493	(5.7)	1.82	(1.71–1.94)*
**Prescribed dispensed medication**						
SSRIs	1793	(16.7)	252,027	(4.5)	3.24	(3.08–3,41)*
TCAs	591	(5.5)	48,405	(0.9)	4.59	(4.22–5.00)*
Other antidepressants	575	(5.3)	95,879	(1.7)	2.55	(2.34–2.78)*
Any antidepressant	2685	(25.0)	363,592	(6.5)	3.64	(3.48–3.80)*
FGA	112	(1.0)	36,626	(0.7)	1.25	(1.04–1.51)
SGA	116	(1.1)	32,307	(0.6)	1.74	(1.45–2.09)*
Any antipsychotic	214	(2.0)	62,075	(1.0)	1.53	(1.33–1.75)*
Benzodiazepines	1134	(10.5)	139,490	(2.5)	3.39	(3.19–3.61)*
Sleeping agents	1324	(12.3)	199,339	(3.6)	2.70	(2.55–2.86)*
Lithium	38	(0.4)	11,383	(0.2)	1.29	(0.94–1.77)
Drugs for alcohol dependance	14	(0.1)	11,716	(0.2)	0.59	(0.35–1.00)
Any psychiatric drug	3801	(35.4)	558,725	(10.1)	3.72	(3.57–3.88)*

a adjusted for age, sex, education level, country of birth, and type of living area, * = significant after Bonferroni-Holm correction for multiple comparisons. SSRI = Selective Serotonin Reuptake Inhibitors, TCA = Tricyclic Antidepressants, FGA = First generation Antipsychotics, SGA = Second generation Antipsychotics.

Regarding psychiatric medication, 35% of the MS patients had been prescribed this compared to 10% of the non-MS individuals (OR 3.72 (95% CI 3.57–3.88)). Twenty-five percent of the MS patients had been prescribed an antidepressant, most commonly SSRIs (17%), compared to 6.5% of the non-MS individuals, (OR 3.64 (95% CI 3.48–3.80)). Ten point five percent of the MS patients had been prescribed benzodiazepines and 12.5% sleeping agents, while the proportions among the non-MS individuals where 2.5% and 3.6%, respectively (ORs 3.39 (95% CI 3.19–3.61) and 2.70 (95% CI 2.55–2.86)). Medication for alcohol dependence was the only drug group less prescribed to MS patients, 0.1% compared to 0.2% among non-MS individuals (OR 0.59 (95% CI 0.35–1.00)).

The results from the survival analysis are shown in table 3. MS patients with any psychiatric diagnosis had a higher HR for disability pension than MS patients without a diagnosis; HR 1.83 (95% CI 1.53–2.18). The diagnosis group with the highest HR for disability pension was personality disorders; HR 5.42 (95% CI 2.98–9.83). MS patients with a depressive disorder diagnosis had a doubled HR for disability pension, HR 1.95 (95% CI 1.54–2.47). HRs were elevated in all diagnostic groups except for bipolar disorder.

**Table 3:** The hazard ratio (HR) with 95% confidence intervals (CI) for being granted disability pension during a five-year follow up (2006–2010) in multiple sclerosis (MS) patients not on disability pension in 2005, n = 4750.

Diagnoses	Events/patients (%)	Adjusted HR^a^ (95% CI)^a^
Developmental, organic disorders	14/24 (0.5)	3.59 (2.11–6.11)*
Substance abuse	19/40 (0.9)	2.20 (1.39–3.47)*
Psychotic disorders	3/11 (0.2)	1.06 (0.34–3.29)
Depressive disorders	75/161 (3.6)	1.95 (1.54–2.47)*
Bipolar disorder	3/10 (0.2)	0.99 (0.32–3.10)
Affective disorders	77/172(3.8)	1.85 (1.47–2.33)*
Neurotic, somatoform disorders	46/113 (2.5)	1.72 (1.28–2.31)*
Stress-related disorders	30/63 (1.4)	2.03 (1.41–2.93)*
Behavioral disorders	9/30 (0.7)	1.23 (0.64–2.37)
Personality disorders	11/12 (0.3)	5.42 (2.98–9.83)*
Any psychiatric diagnosis	144/342 (7.6)	1.83 (1.53–2.18)*
**Prescribed dispensed medication**		
SSRIs	207/415 (9.2)	2.15 (1.85–2.49)*
TCAs	61/123 (2.7)	2.04 (1.58–2.63)*
Other antidepressants	74/155 (3.4)	2.02 (1.60–2.56)*
Any antidepressant	309/632 (14.0)	2.23 (1.96–2.54)*
FGA	8/15 (0.3)	2.48 (1.23–4.99)
SGA	6/13 (0.3)	2.37 (1.06–5.30)
Any antipsychotic	13/26 (0.6)	2.56 (1.48–4.44)*
Benzodiazepines	74/168 (3.7)	1.77 (1.40–2.24)*
Sleeping agents	138/320 (7.1)	1.73 (1.45–2.07)*
Lithium	3/9 (0.2)	1.11 (0.36–3.44)
Drugs for alcohol dependance	1/2 (0.0)	1.30 (0.89–1.89)
Any psychiatric drug	403/900 (19.9)	2.09 (1.84–2.33)*

a adjusted for age, sex, education level, country of birth and type of living area. * = significant after Bonferroni-Holm correction for multiple comparisons. SSRI = Selective Serotonin Reuptake Inhibitors, TCA = Tricyclic Antidepressants, FGA = First generation Antipsychotics, SGA = Second generation Antipsychotics.

MS patients with a psychiatric drug prescription of any kind had a higher HR for disability pension, HR 2.09 (95% CI 1.84–2.33) than MS patients without a prescription. The drug group with the highest HR was antipsychotics, HR 2.56 (95% CI 1.48–4.44). Those prescribed SSRIs, the most common group, had a doubled HR for disability pension, HR 2.15 (95% CI 1.85–2.49). All separate drug groups were associated with an elevated risk.

## Discussion

### Main findings

To the best of our knowledge, this study is the first to examine the distribution of specific psychiatric diagnoses as well as medications among MS patients of working age, and their association with future disability pension. MS patients of working age had an elevated risk for being diagnosed with most types of psychiatric disorders, as well as being prescribed most types of psychiatric medications compared to non-MS individuals. Moreover, being diagnosed with a psychiatric disorder or having been prescribed psychiatric medication generally meant an elevated risk for future disability pension among MS patients.

### Strengths and limitations

The strengths of this study include the use of data from several nationwide registers of high quality as well as the use of a large nationwide cohort. Missing data was relatively uncommon and there were no loss to follow-up. All Swedish MS patients of working age found in our registers were included in the cohort, which granted sufficient statistical power to analyze most psychiatric diagnoses and medications in detail.

There are also limitations that need to be addressed. First, the method of identifying MS patients means that patients who had not received any specialty care during the five-year interval studied were misclassified. Second, the number of patients with some of the more uncommon psychiatric diagnoses and/or medications was quite low, especially in the survival analysis, which led to wide 95% CIs. Third, pharmacological data only included recipes dispensed in pharmacies during July to December 2005, which means that medication not dispensed during this time was misclassified. Fourth, psychiatric diagnoses given in a primary care setting were not included. While it is likely that many MS patients receive a psychiatric diagnosis from their neurologist in specialty care, a higher rate of the non-MS individuals probably receive their psychiatric diagnoses from a general practitioner. This may lead to a differential misclassification and a skewed comparison regarding psychiatric diagnoses often treated in primary care such as mild to moderate depression and anxiety disorders. On the other hand, in the Swedish health care system, only the registration of a primary diagnosis is mandatory. It is possible that specialty care practitioners, e.g. neurologists, do not specifically register a second or third diagnosis during in- or outpatient care given to MS patients, although psychiatric symptoms may be present. With this in mind, the rate of psychiatric comorbidity could be higher among MS patients than these data reflect. This could be supported by the consistently higher rates in this study of psychiatric medications prescribed compared to their responding diagnoses. This also highlights the question as to whether psychiatric symptoms in MS should be considered comorbid rather than symptoms inherent to the disease itself, an important question both in an epidemiological [29] and clinical [30] context.

### Comparison with previous studies

This is, to the best of our knowledge, the first study examining the association of specific psychiatric diagnoses and medications with disability pension among MS patients. It is also the first study comparing the use of psychiatric medications with that of non-MS individuals. Thus, comparisons with previous studies can be made mainly regarding the prevalence of psychiatric diagnoses. Regarding the method of identifying MS and psychiatric comorbidity using administrative data, Marrie et al recently published a validation study using a combined diagnosis and medication model [20]. In this study we chose to analyze diagnoses and medications separately, rather than constructing models of presumed clinical validity.

Depressive disorder was, as expected, the most common psychiatric diagnosis among both MS patients and non-MS individuals of working age. The 4.4% of MS patients who had been diagnosed with depression during the five years studied is a rather low proportion compared with previous studies [13]–[15]. However, most previous estimates of prevalence of psychiatric diagnoses are based on survey or clinical studies, which seem to lead to higher estimates. The exclusion of patients over 65 years of age is another possible contributing factor. The risk for bipolar disorder was nearly doubled in MS patients compared to non-MS individuals, but the prevalence of 0.5% was lower than in other studies [15], [20]. The risk for obtaining a psychotic disorder diagnosis tended to be elevated in MS patients with a 0.8% prevalence, in line with the only previous register study on this topic [22].

### Implications of results

In this study, 35% of all MS patients received a psychiatric medication during the six months observed, which was a high number compared to non-MS individuals. Some of these drugs are also used to treat neurological symptoms, e.g. TCAs and duloxetine for neuropathic pain, SSRIs and TCAs for pseudobulbary affect, and benzodiazepines for spasticity. However, that the most commonly prescribed drug class in the study, SSRIs, was prescribed to 17% of MS patients suggests a high prevalence of depressive disorder and anxiety disorders. Of note is that previous studies have shown that depression is generally underdiagnosed and undertreated in MS patients [17], [31], which indicates that the prescription rates should perhaps be even higher if MS patients were to receive adequate antidepressant treatment.

In the survival analysis, psychiatric diagnoses and medications were generally associated with a substantially higher HR for disability pension. Depression was the diagnosis associated with the highest number of patients being granted disability pension. It is possible that this is due to a causative effect of depression, but could also be interpreted as a psychosocial reaction to an increased general disability eventually leading to disability pension. Coping mechanisms have been shown to mediate the relation between depression and disability [32], which suggests that psychosocial interventions could have an preemptive effect on the risk for disability pension in this group of patients, and suggesting depression as a target area for improved diagnosis and management in MS patients.

Personality disorders were the diagnoses with the highest HR for disability pension, which could be mediated by coping difficulties often present in personality disorders [33], but also by the fact that cognitive impairment is overrepresented in this group [34], The diagnosis group of organic and developmental disorders, which includes neuropsychiatric conditions considered to be secondary to an organic disease, also increased the HR for disability pension substantially. This group is heterogeneous, but its effect on the HR for disability pension probably reflects the disabling effects of conditions associated with latter stages of MS disease such as pseudobulbar affect and euphoria sclerotica.

Among the medications, antipsychotics were associated with the highest HR for disability pension, reflecting the serious nature of the conditions often treated with this class of drugs, namely bipolar disease and psychotic disorders. Additionally, the side effect profile of these medications, including various anticholinergic effects and extrapyramidal symptoms, could be especially unwanted in MS patients, and a causal contributing effect cannot be ruled out. MS patients had a risk three times that of the non-MS individuals for being prescribed benzodiazepines, which could, at least partially, be explained by treatment of spasticity, but most likely reflect high rates of anxiety disorders and sleeping problems. These rates must be considered high as modern guidelines do not recommend benzodiazepines at all as treatment for anxiety disorders due to concerns of tolerance and dependence [35], and the evidence for treatment of spasticity is at best uncertain [36]. Even more MS patients, 12%, had been prescribed short-acting sleeping agents. Although 40% of MS patients reportedly suffer from different kinds of insomnia [37], short-term sleeping agents are generally recommended for short-time use only [38], due to concerns of tolerance and dependence [39]. In general, any health problem for which antidepressants or sleep agents are prescribed, be it psychiatric disease, insomnia for non-psychiatric reasons, neuropathic pains or spasticity confer additional burden for patients with MS and may thus contribute to the risk of sick leave and disability pension. Possible contributing effects of the medications themselves on risk for disability pension should, however, not be ruled out.

An interesting finding is that only 0.1% of all MS patients in this study had been prescribed medication for alcohol abuse, less than in non-MS individuals. The one-month prevalence of alcohol dependence in MS patients has been reported to be 14%, and to be more common in working patients [40]. In this study, a diagnosis of a substance related disorder more than doubled the risk for disability pension, again suggesting an area for improved clinical detection and management.

### Conclusion

In this population-based register study, MS patients of working age had an elevated risk for most psychiatric diagnoses and medications compared to non-MS individuals. Furthermore, MS patients having a psychiatric diagnosis or medication were at higher risk for future disability pension than MS patients who were not. These findings highlight the need for, and importance of, correct diagnosis and management of psychiatric comorbidity in MS patients. Suggestions for further research include investigations into what extent MS patients actually do receive accurate psychiatric diagnoses and optimal care, prescription patterns of psychiatric drugs in MS patients, and if intensified management of psychiatric comorbidity could decrease the risk for future disability pension and other adverse outcomes.
